# Associations between Sedentary Time and Sedentary Patterns and Cardiorespiratory Fitness in Chinese Children and Adolescents

**DOI:** 10.3390/children9081140

**Published:** 2022-07-29

**Authors:** Ming Li, Xiaojian Yin, Yuqiang Li, Yi Sun, Ting Zhang, Feng Zhang, Yuan Liu, Yaru Guo, Pengwei Sun

**Affiliations:** 1 College of Physical Education and Health, East China Normal University, Shanghai 200241, China; liming23416@163.com (M.L.); liyuqiang-123@126.com (Y.L.); sunyi0084@163.com (Y.S.); noway1982@163.com (T.Z.); fzhang1988@126.com (F.Z.); yliu0809@163.com (Y.L.); yaruguo3299@163.com (Y.G.); 877523197@163.com (P.S.); 2 Key Laboratory of Adolescent Health Assessment and Exercise Intervention of Ministry of Education, East China Normal University, Shanghai 200241, China; 3 College of Economics and Management, Shanghai Institute of Technology, Shanghai 201418, China

**Keywords:** sedentary time, sedentary pattern, physical activity, cardiorespiratory fitness

## Abstract

The increase in sedentary behavior in children and adolescents has become a worldwide public health problem. This study aimed to explore the associations between sedentary time (ST) and sedentary patterns (SP) and the cardiorespiratory fitness (CRF) of Chinese children and adolescents. The CRF of 535 participants was determined using a 20-m shuttle run test. ST and SP were measured with accelerometers. Questionnaires were used to investigate the different types of ST. Multiple linear regression models were used to test the associations between ST and SP and CRF. In this study, only some ST and SP indicators were found to be significantly associated with CRF in girls. With each additional 10 min of screen time or passive traffic time, VO_2max_ decreases by 0.06 mL/kg/min (*B* = −0.006, 95% *CI*: −0.010~−0.001) and 0.31 mL/kg/min (*B* = −0.031, 95% *CI*: −0.061~−0.002), respectively, with MVPA control. With each additional 10 min of breaks in ST or duration of breaks in ST, VO_2max_ increases by 0.41 mL/kg/min (*B* = 0.041, 95% *CI*: 0.007~0.076) and 0.21 mL/kg/min (*B* = 0.021, 95% *CI*: 0.007~0.035), respectively, with control total ST. Breaks in ST (*B* = 0.075, 95% *CI*: 0.027~0.123) and the duration of breaks in ST (*B* = 0.021, 95% *CI*: 0.012~0.146) were positively correlated with CRF when controlling for LPA, but these associations were not significant when controlling for MVPA (*B* = 0.003, 95% *CI*: −0.042~0.048; *B* = 0.001, 95% *CI*: −0.024~0.025). The total ST of children and adolescents was found to not be correlated with CRF, but when ST was divided into different types, the screen time and passive traffic time of girls were negatively correlated with CRF. More breaks in ST and the duration of breaks in ST were positively associated with higher CRF in girls. MVPA performed during breaks in ST may be the key factor affecting CRF. Schools and public health departments should take all feasible means to actively intervene with CRF in children and adolescents.

## 1. Introduction

Cardiorespiratory fitness (CRF) reflects the ability of the human body to absorb, transport, and utilize oxygen, which is the core element of physical health [1]. Many studies have confirmed that low CRF is highly associated with cardiovascular disease and all-cause mortality [2]. In recent decades, the levels of CRF of children and adolescents in the world have shown a continuous downward trend [3]. Therefore, urgent effective measures to improve CRF should be taken. Increasing physical activity, especially moderate-to-vigorous physical activity (MVPA), is an effective way to improve CRF in children and adolescents [4,5]. However, MVPA only accounts for a minor part of the waking time of children and adolescents every day; most of the day includes sedentary time (ST) [6]. Exploring the association between ST and CRF may lead to new breakthroughs for improving CRF in children and adolescents.

Sedentary behavior is any waking behavior characterized by an energy expenditure ≤1.5 METs while in a sitting or reclining position [7]. ST can be divided into screen time and non-screen time based on whether it is related to electronic screens [8]. For children and adolescents, screen time mainly includes watching TV/movies, using computers to play games, using mobile phones/tablets, etc. Non-screen time mainly includes attending class, writing extracurricular assignments, reading extracurricular books, attending extracurricular tutoring, passive traffic time, social sedentary time, etc. [8]. A previous study suggested that excessive ST may be an important risk factor for low CRF in children and adolescents [9]. However, the relationships between different types of ST and CRF in children and adolescents vary [7]. In order to reduce the health hazards caused by ST, an effective solution is to reduce the specific types of ST that cause health risks as much as possible [10]. However, most existing studies focus on total ST or a certain type of ST, which does not offer insights into whether a specific type of ST or total ST affects CRF. In addition, the relationship between the component characteristics of ST and CRF also deserves further consideration. The total ST measured by an accelerometer includes all components of ST (prolonged ST or non-prolonged ST). The relationship between different components of ST and CRF may not be consistent [11]. Therefore, further study is needed to comprehensively consider the impact of ST on CRF in children and adolescents from two aspects, type and component.

Along with ST, sedentary patterns (SP) may also be relevant for CRF [12]. SP reflects the manner of ST accumulation, which can be expressed by sedentary bouts, breaks in ST, and the duration of breaks in ST. The existing research results on the relationship between SP and CRF are not consistent. For example, Júdice et al. found that breaks in ST and non-prolonged sedentary bouts were positively associated with CRF [11]. However, Bailey et al. [13] and Denton et al. [14] did not find that breaks in ST were significantly associated with CRF. Furthermore, existing studies do not suggest which duration of breaks in ST is most beneficial for CRF.

In summary, this study selected representative nationwide samples from China, adopted a combination of subjective and objective methods to comprehensively analyze the associations between ST and SP and CRF in children and adolescents, and further identified the duration of breaks in ST that are beneficial to CRF, providing a reference for schools and public health departments to develop targeted interventions.

## 2. Materials and Methods

### 2.1. Participants

From March to July 2019, the research team recruited 840 children and adolescents aged 7–18 for the study. The children and adolescents came from seven Chinese cities: Shanghai, Taiyuan, Guangzhou, Changsha, Urumqi, Chengdu and Kunming. The research team randomly selected one elementary school, one middle school, and one high school in each city as study sites. Five boys and five girls were randomly selected from each age group. All students can complete a CRF test. 120 students were excluded for failing to wear accelerometers, and 185 were excluded for not completing the 20-m shuttle run test (SRT). A total of 535 students were finally included in this study.

### 2.2. CRF

The research team tested 20-m SRT in all participants. After warming up, participants stood on one of two horizontal lines placed 20 m apart. The participants, running back and forth between the lines, were required to increase their speed according to the music. The initial speed was 8.0 km/h, the second level was 9.0 km/h, and then the running speed was increased by 0.5 km/h for each level. When the participants could not maintain the speed set by the music, or could not follow the music’s rhythm to reach the end line two consecutive times, the test was terminated. The total number was recorded as the final score [15]. Matsuzaka et al.’s [16] formula was used to estimate the maximum oxygen uptake: VO_2max_ = 61.1 − 2.20 × gender − 0.462 × age − 0.862 × BMI + 0.192 × laps, where gender is expressed as 0 for boys and 1 for girls. VO_2max_ is usually used to reflect CRF, and 20-m SRT is highly correlated with VO_2max_ [17]. Therefore, we can use 20-m SRT to evaluate CRF in children and adolescents.

### 2.3. Sedentary Behavior

In this study, a subjective and objective approach was used to measure sedentary behavior (SB). Total ST, prolonged ST or non-prolonged ST, and SP were objectively measured using a GT3X+ (ActiGraph, Pensacola, FL, USA) accelerometer. During the measurement, the subjects were told to wear the accelerometer on the right hip for seven consecutive days—including five school days and two weekend days—and that it could not be removed except for bathing, swimming, and sleeping. The epoch duration was set at 5 s. The accelerometer began to record data from the early morning of the second day, and was retrieved by the researcher on the eighth day. The original data were downloaded through ActiLife version 6.10.2 software (ActiGraph, Pensacola, FL, USA). After the original data were downloaded, their validity was first screened: valid data needed to include at least three valid school days and one valid weekend day. A valid school day or weekend day meant at least 10 h of the device being effectively worn on the test day [18]. The cut-points of Evenson et al. [19] were adopted to classify SB (0–100 counts/min), light physical activity (LPA) (101–2295 counts/min), moderate physical activity (MPA) (2296–4011 counts/min), and vigorous physical activity (VPA) (4012 counts/min or more). These cut-points have high validity and reliability in the evaluation of the PA of children and adolescents [20]. The total ST was divided into prolonged ST (at least 20 min uninterrupted ST) and non-prolonged ST (less than 20 min ST). An experimental study has shown that children and adolescents who often sit for more than 20 min are at risk of metabolic diseases [21]. Based on this, 20 min was chosen as the cut-off point for distinguishing between prolonged ST and non-prolonged ST. The SP were expressed by prolonged sedentary bouts (the number of instances of at least 20 min uninterrupted ST), non-prolonged sedentary bouts (the number of instances of less than 20 min uninterrupted ST), breaks in ST (the number of interruptions in ST in which the accelerometer count raised above 100 counts/min, and which stayed for at least 1 min), and the duration of breaks in ST (the total time of breaks in ST). A questionnaire was used to measure the duration of the different types of ST. The questionnaire for children aged 7–9 was filled out with the help of their parents. Participants reported how much time they spent doing the following activities: watching TV/movies, using computers to play games, using mobile phones/tablets (screen time) from Monday to Friday and on Saturday and Sunday; writing extracurricular assignments, reading extracurricular books, attending extracurricular tutoring (these three behaviors are defined as learning behaviors that occur outside of class, and are collectively referred to as extracurricular learning time); sitting and chatting (social ST); and commuting to school (passive traffic ST). The time measured in the above questionnaire was the weighted average on school days (5/7) and the weekend (2/7). The test–retest reliability of each item in the questionnaire was between 0.79–0.91, which is acceptable.

### 2.4. Covariate

Urban or rural residence, socioeconomic status (SES), sleep time, and BMI were potential confounders. Questionnaires were conducted to collect information on urban or rural residence, SES, and sleep time. Parental education, parental occupation, and household income were used to assess the SES [22]. The sleep time duration, from the time of going to bed at night to getting up in the morning, was filled out by the individual. Body height was determined using a mechanical height gauge, and body weight was measured using an electronic scale. The values were accurate to 1 decimal place.

### 2.5. Statistics Analysis

The normality of all variables was tested using a histogram and Q–Q plot. An independent sample *t*-test (for normalvariables) and Mann–Whitney *U* test (for skewedvariables) were used to compare the gender differences of each variable. A chi-squared test was used to compare the gender differences of residence. Spearman’s correlation was used to test the correlations between various variables. Multiple linear regression models were used to test the associations between different types of ST and SP and CRF in children and adolescents. In the analysis of the relationship between CRF and ST, CRF was included in the model as the dependent variable, and ST indexes as the independent variable. Model 1 adjusted for the age, urban and rural areas, SES, sleep time, and BMI; Model 2 further adjusted for the MVPA based on Model 1. In the analysis of the relationship between CRF and SP, CRF was included in the model as the dependent variable, and SP indexes as the independent variable. Model 1 adjusted for the age, urban and rural areas, SES, sleep time, and BMI; Model 2 further adjusted for the total ST based on Model 1. In the analysis of the intensity attributes of the duration of breaks in ST, CRF was included in the model as the dependent variable, and breaks in ST or the duration of breaks in ST as the independent variable. Model 1 adjusted for the age, urban and rural areas, SES, sleep time, BMI, total ST, and LPA; Model 2 adjusted for the age, urban and rural areas, SES, sleep time, BMI, total ST, and MVPA. Statistical significance was set at 0.05, and all analyses were conducted using IBM SPSS version 25.0 for Windows (IBM Corp., Armonk, NY, USA).

## 3. Results

### 3.1. Descriptive Characteristics of the Sample

Table 1 presents the descriptive characteristics of the sample, including demographic, CRF, and MVPA data. A total of 535 participants (47.8% boys, 52.2% girls) were included in this study. The table shows that the BMI, sleep time, VO_2max_, and MVPA values of boys were higher than those of girls, and the difference was statistically significant (*p* < 0.05).

**Figure 1:**
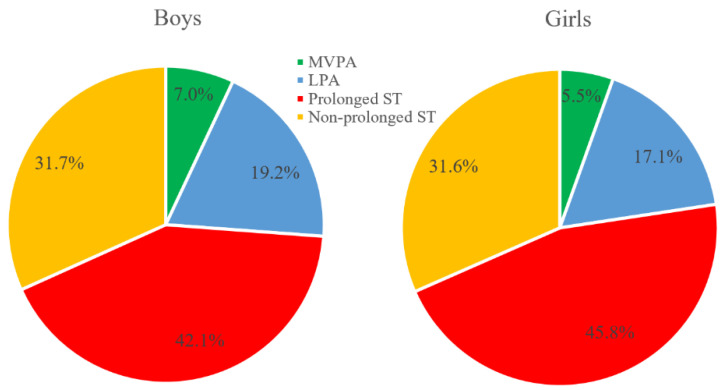
The proportions of prolonged ST, non-prolonged ST, LPA, and MVPA, stratified by gender.

Figure 1 presents that the proportion of prolonged ST (42.1%) of boys was lower than that of girls (45.8%), the proportions of MVPA (7.0%) and LPA (19.2%) were higher than those of girls (5.5% and 17.1%), and the proportion of non-prolonged ST (31.7%) was close to that of girls (31.6%).

### 3.2. Descriptive Characteristics of ST and SP

Table 2 presents the descriptive characteristics of the different types of ST and SP. In the comparison of ST, the total ST (655.1 min/d) and prolonged ST (373.7 min/d) of boys were higher than those of girls (699.5 min/d and 414.0 min/d), and the difference was statistically significant (*p* < 0.001). There were no significant gender differences for other types of ST. In the comparison of SP, non-prolonged sedentary bouts (30.0 number/d) and breaks in ST (41.2 number/d) for boys were higher than those for girls (22.8 number/d and 33.7 number/d), and the difference was statistically significant (*p* < 0.001). There was no significant sex difference in the number of prolonged sedentary bouts.

### 3.3. Correlation Analysis of SB Variables and CRF

Table 3 shows that the VO_2max_ values were positively associated with non-prolonged sedentary bouts (*r* = 0.17, *p* < 0.01), breaks in ST (*r* = 0.19, *p* < 0.01), and the duration of breaks in ST (*r* = 0.19, *p* < 0.01).

### 3.4. Associations between ST and CRF in Children and Adolescents

Table 4 shows that there were negative associations between screen time (*B* = −0.005, 95% *CI*: −0.010~−0.001, adjusted *R*^2^ = 0.59) and passive traffic ST (*B* = −0.030, 95% *CI*: −0.061~−0.001, adjusted *R*^2^ = 0.62) and CRF in girls, and the associations were still significant after further controlling for MVPA (*B* = −0.006, 95% *CI*: −0.010~−0.001, adjusted *R*^2^ = 0.61; *B* = −0.031, 95% *CI*: −0.061~−0.002, adjusted *R*^2^ = 0.64). This means that for each additional 10 min of screen time or passive traffic time, VO_2max_ decreases by 0.06 mL/kg/min and 0.31 mL/kg/min, respectively, with MVPA control. There was no statistically significant association between ST and CRF in boys.

### 3.5. Associations between SP and CRF in Children and Adolescents

Table 5 shows that there were no statistically significant association between breaks in ST (*B* = 0.031, 95% *CI*: −0.003~0.064) and CRF in girls, but these were positively correlated after controlling for total ST (*B* = 0.041, 95% *CI*: 0.007~0.076, adjusted *R*^2^ = 0.59). Further, the duration of sedentary breaks (*B* = 0.015, 95% *CI*:0.001~0.028, adjusted *R*^2^ = 0.59) was positively associated with CRF, and the association was still significant after further controlling for the total ST (*B* = 0.021, 95% *CI*:0.007~0.035, adjusted *R*^2^ = 0.60). This means that for each additional 10 min of breaks in ST or the duration of breaks in ST, VO_2max_ increases by 0.41 mL/kg/min and 0.21 mL/kg/min, respectively, with control total ST. There was no statistically significant association between SP and CRF in boys.

### 3.6. Exploration of the Intensity Attribute of the Duration of Breaks in ST in Girls

Table 6 shows that breaks in ST (*B* = 0.075, 95% *CI*: 0.027~0.123) and the duration of breaks in ST (*B* = 0.021, 95% *CI*:0.012~0.146) were positively correlated with CRF when controlling for LPA in girls, but these associations were not significant when controlling for MVPA (*B* = 0.003, 95% *CI*: −0.042~0.048; *B* = 0.001, 95% *CI*: −0.024~0.025). This means that MVPA in the duration of breaks in ST is a key component affecting CRF.

## 4. Discussion

The purpose of this study was to explore the associations between different types of ST and SP and CRF in children and adolescents. The study found that there was no significant correlation between total ST and CRF in children and adolescents, but when ST was divided into different types, the screen time and passive traffic ST of girls were negatively correlated with CRF and independent of MVPA. Breaks in ST and the duration of breaks in ST were positively correlated with CRF in girls. MVPA performed during breaks in ST may be the key factor affecting CRF.

Most studies have confirmed that MVPA is positively correlated with CRF in children and adolescents [4,5]. However, in view of the extremely low proportion of MVPA performed in a day, exploring the relationship between ST and CRF may provide new breakthroughs for improving CRF in children and adolescents. For example, Santos et al. found that the effect of ST on CRF was independent of physical activity, and even an additional increase in MVPA could not offset the adverse effect of prolonged ST on CRF over a long period of time. Reducing ST is an important means to improve CRF in children and adolescents [23]. Other studies have also found a negative correlation between ST and CRF, but the difference in their findings was that increasing MVPA was found to be able to weaken [9,24] or offset [25] the adverse effect of ST on CRF. However, some studies did not find a significant correlation between ST and CRF in children and adolescents [14,26,27]; the results of the present study also support this conclusion. In addition, after further dividing the total ST into prolonged ST and non-prolonged ST, this study found that distinguishing between the components of ST (prolonged ST and non-prolonged ST) had no effect on the significance of the association. The inconsistency of research conclusions described here may be related to the inconsistency of the cut-points of sedentary behavior in different studies, resulting in the incomparability of ST [14]. Therefore, in follow-up studies, when investigating the relationship between ST and CRF in children and adolescents, the cut-off point of sedentary behavior should include the widely used and reliable boundary value, so as to facilitate the horizontal comparison of different studies. It is important to note that although this study and previous studies did not find a significant association between the duration of sedentary breaks and CRF in children and adolescents, there is evidence that higher levels of ST are negatively associated with health outcomes in adults [28]. Therefore, we cannot ignore the possible long-term adverse effects of ST.

Due to the popularity of portable devices with electronic screens, the screen time of children and adolescents has shown a rapid upward trend in recent decades [10]. Most studies support the theory that screen time is negatively correlated with CRF, and several studies have indicated that more than 2 h of screen time per day is related to a decline in CRF. Consistent with previous studies, this study found that although the association between total ST and CRF was not significant, when ST was divided into different types, the screen time of females was negatively correlated with CRF and independent of MVPA. It should be noted that the screen time investigated in this study was only recreational ST. In recent years, children and adolescents’ learning ST has been increasing due to the widespread use of multimedia technology in daily teaching and the online teaching methods adopted during the COVID-19 pandemic [10]. The sedentary guidelines of existing countries clearly propose that online teaching time should be limited to avoid possible health threats caused by excessive screen time [8]. However, the current evidence on the relationship between learning ST and CRF in children and adolescents is still unclear. Future research should further distinguish the nature of ST, and accurately identify risk factors for CRF.

Previous studies have focused on the relationship between active transportation to school and CRF, and most studies support the theory that active transportation, especially cycling to school, is positively correlated with CRF in children and adolescents [29]. Other studies have confirmed that the CRF of children and adolescents who travel to school by passive transportation is lower than that of individuals who travel to school by active transportation [30]. The findings of this study further improve the chain of evidence that suggests that passive traffic-sitting in girls is negatively correlated with CRF and independent of MVPA. Based on the above evidence chain, we can speculate that children and adolescents who travel to school by active transportation have higher CRF, whereas those who travel to school by passive transportation have lower CRF. The reason for this may be that active transportation to school increases physical activity, which is positively correlated with CRF, whereas passive transportation increases ST, which is negatively correlated with CRF.

This study did not find a significant association between extracurricular learning ST and CRF. Although there is no clear evidence that too much extracurricular learning ST has a detrimental effect on CRF, given the negative impact of too much extracurricular learning ST on other movement behaviors [10] and the positive correlation between movement behavior and CRF [31], we still cannot ignore the potential long-term adverse effects of a heavy schoolwork load on CRF in children and adolescents. Moreover, this study found that the extracurricular learning ST of Chinese children and adolescents (about 360 min/d) is much higher than the recommended amount of international school-based sedentary behavior recommendations (10~60 min/d) [8]. Reducing the schoolwork load of Chinese children and adolescents should be targeted.

This study found that breaks in ST and the duration of breaks in ST were positively associated with CRF in girls. Combined with the high correlation between breaks in ST and the duration of breaks in ST (r = 0.86), we can speculate that more breaks in ST can lead to longer durations of breaks in ST, which may be a key factor in improving CRF in children and adolescents. This result also confirms previous research findings. Judice et al.’s study showed that the benefit of sedentary bout interruptions did not come from the interruptions themselves, but from the physical activity performed during the sedentary bout interruptions [11]. The results of this study suggest that breaks in ST can have a beneficial effect on CRF. Breaks in ST are essentially a change from sedentary behavior to physical activity. Intensity is an important aspect to consider. This study found that breaks in ST and the duration of breaks in ST were positively associated with CRF after further controlling for LPA. However, the association was not significant after controlling for MVPA. We can speculate that in this study, MVPA was the determinant factor affecting CRF in the accumulated duration of breaks in the ST of girls. The results of this study suggest that although directly increasing MVPA is an important means to promote CRF, considering the generally low MVPA levels of children and adolescents [32], increasing MVPA by increasing breaks in ST and the duration of breaks in ST may also be a feasible method to improve CRF. The latest release of the International School-based Sedentary Behavior Recommendations also suggests that prolonged ST should be interrupted as much as possible to reduce the harm of sedentary behavior to children and adolescents [8].

We further evaluated the effects of screen time, passive traffic time, breaks in ST, and the duration of breaks in ST on the goodness-of-fit of the models using a hierarchical regression method. The results showed that the adjusted *R*^2^ was increased by 0.012, 0.016, 0.009, and 0.014, respectively (Supplementary Materials). Although the above significant variables had little influence on the goodness-of-fit of the model, we still need to pay attention to them as an important means of CRF intervention in children and adolescents. Previous studies pointed out that although MVPA or ST showed low-to-medium correlations with CRF in children and adolescents, it did not mean that they were not important [5,7]. Therefore, schools and public health departments should take all feasible means to actively intervene with CRF in children and adolescents.

## 5. Strengths and Limitations

A major strength of this study was that it selected a representative sample from six administrative regions in China. Furthermore, this study adopted a combination of objective and subjective measures to evaluate sedentary behavior, which is conducive to a more comprehensive understanding of the relationship between the ST and SP and CRF in children and adolescents. There are still several limitations which should be considered. First, as this was a cross-sectional study, it was impossible to determine the continuous impact of ST and SP on CRF. Second, the ST objectively measured using an accelerometer in this study may have been overestimated, as lower-intensity behaviors, such as standing or lying down, may have been identified as sedentary behavior.

## 6. Conclusions

There was no significant correlation between total ST and CRF in children and adolescents, but when ST was divided into different types, the screen time and passive traffic ST of girls were negatively correlated with CRF and independent of MVPA. Breaks in ST and the duration of breaks in ST were positively correlated with CRF in girls. MVPA performed during breaks in ST may be the key factor affecting CRF. Schools and public health departments should take all feasible means to actively intervene with CRF in children and adolescents.

## Figures and Tables

**Table 1:** Descriptive characteristics of the sample.

Parameters	Boys (*n* = 255)	Girls (*n* = 280)	*p*	Cohen’s *d*
Age (y) ^a^	12.4 ± 3.4	12.7 ± 3.3	0.20	0.09
Residence (%) ^b^				
Urban	61.2	65.4	0.32	0.04
		
Rural	38.8	34.6		
Height (cm) ^a^	153.2 ± 19.7	150.6 ± 15.2	0.08	0.15
Weight (kg) ^a^	45.3 ± 17	41.7 ± 12.3	<0.001	0.24
BMI (kg/m^2^) ^a^	18.6 ± 3.2	18.0 ± 2.9	0.02	0.20
SES ^a^	0.05 ± 1.1	−0.03 ± 0.9	0.42	0.08
Sleep time (h/d) ^a^	8.4 ± 1.3	8.0 ± 1.6	<0.001	0.28
VO_2max_ (mL/kg/min) ^a^	46.5 ± 4.1	42.9 ± 3.6	<0.001	0.93
MVPA (min/d) ^a^	62.0 ± 19.3	49.5 ± 15.5	<0.001	0.71

Note: Values are presented as mean ± SD and percent; ^a^ independent sample *t*-test; ^b^ chi-squared test. BMI, body mass index; SES, socioeconomic status; MVPA, moderate-to-vigorous physical activity.

**Table 2:** Descriptive characteristics of ST and SP.

Parameters	Boys (*n* = 255)	Girls (*n* = 280)	*p*	Cohen’s *d*
Total ST (min/d) ^a^	655.1 ± 119.9	699.5 ± 106.7	<0.001	0.30
Prolonged ST (min/d) ^a^	373.7 ± 157.4	414.0 ± 145.4	<0.001	0.27
Non-prolonged ST (min/d) ^a^	281.5 ± 92.3	285.5 ± 94.3	0.62	0.04
Screen time (min/d) ^b^	31.4 (4.29, 88.9)	30.0 (2.86, 95.7)	0.81	0.02
Extracurricular learning time (min/d) ^a^	350.7 (259.3, 490.7)	345.7 (250.7, 500.0)	0.99	<0.01
Passive traffic ST (min/d) ^a^	10.7 (5.7, 22.9)	12.1 (7.1, 17.4)	0.97	<0.01
Social ST (min/d) ^a^	22.9 (12.9, 51.3)	22.9 (15.2, 42.9)	0.64	0.05
Prolonged sedentary bouts (number/d) ^a^	10.1 ± 3.2	9.9 ± 3.0	0.43	0.06
Non-prolonged sedentary bouts (number/d) ^a^	30.0 ± 14.2	22.8 ± 12	<0.001	0.55
Breaks in ST (number/d) ^a^	41.2 ± 13.8	33.7 ± 11.5	<0.001	0.59
Duration of breaks in ST (min/d) ^a^	98.1 ± 31.1	78.3 ± 24.0	<0.001	0.71

Note: Values are presented as mean ± SD and median (25th, 75th percentiles). ^a^ independent sample *t*-test; ^b^ Mann–Whitney *U* test. ST, sedentary time.

**Table 3:** Correlation analysis of each variable.

Parameters	1	2	3	4	5	6	7	8	9	10	11	12
1. VO_2max_	-											
2. Total ST	−0.09	-										
3. Prolonged ST	0.05	0.07	-									
4. Non-prolonged ST	−0.06	−0.05	−0.83 **	-								
5. Screen time	−0.04	−0.06	−0.05	0.07	-							
6. Extracurricular learning ST	−0.06	0.19 **	−0.03	−0.01	−0.05	-						
7. Passive traffic ST	−0.13	−0.07	−0.14 *	0.11	0.07	0.03	-					
8. Social ST	−0.001	−0.08	0.03	−0.04	0.24 **	−0.01	−0.04	-				
9. Prolonged sedentary bouts	0.03	0.06	0.91 **	−0.73 **	−0.05	−0.04	−0.16 *	0.06	-			
10. Non-prolonged sedentary bouts	0.17 **	−0.62 **	−0.24 **	0.16 **	−0.02	−0.14 **	−0.05	−0.01	−0.25 **	-		
11. Breaks in ST	0.19 **	−0.62 **	−0.03	−0.01	−0.04	−0.15 **	−0.10	0.001	−0.02	0.97 **	-	
12. Duration of breaks in ST	0.19 **	−0.51 **	−0.05	0.02	0.07	−0.17 **	−0.09	0.05	−0.05	0.84 **	0.86 **	-

Note: ST, sedentary time. * *p* < 0.05, ** *p* < 0.01.

**Table 4:** Associations between ST and CRF in children and adolescents.

Independent Variables	Boys		Girls	
	Model 1	Model 2	Model 1	Model 2
Total ST	−0.001 (−0.005, 0.004)	0.001 (−0.004, 0.005)	0.003 (−0.001, 0.007)	0.005 (−0.001, 0.009)
Prolonged ST	−0.001 (−0.003, 0.002)	−0.001 (−0.002, 0.002)	0.001 (−0.001, 0.003)	0.001 (−0.001, 0.003)
Non-prolonged ST	−0.001 (−0.005, 0.004)	−0.001 (−0.005, 0.004)	−0.001 (−0.004, 0.002)	−0.001 (−0.004, 0.002)
Screen time	−0.002 (−0.007, 0.003)	−0.002 (−0.008, 0.003)	−0.005 (−0.010, −0.001) *	−0.006 (−0.010, −0.001) **
Extracurricular learning ST	0.001 (−0.002, 0.003)	0.001 (−0.001, 0.003)	−0.001 (−0.003, 0.001)	−0.001 (−0.002, 0.001)
Passive traffic ST	0.001 (−0.033, 0.035)	0.001 (−0.033, 0.035)	−0.030 (−0.061, −0.001) *	−0.031 (−0.061, −0.002) *
Social ST	0.004 (−0.006, 0.015)	0.004 (−0.007, 0.015)	0.003 (−0.004, 0.010)	0.003 (−0.003, 0.010)

Note: The values are presented as *B* (95% *CI*). Model 1 adjusted for age, urban and rural areas, SES, sleep time, and BMI; Model 2 adjusted for Model 1 + MVPA. CRF, cardiorespiratory fitness; BMI, body mass index; SES, socioeconomic status; ST, sedentary time. * *p* < 0.05, ** *p* < 0.01.

**Table 5:** Associations between sedentary patterns and CRF in children and adolescents.

Independent Variables	Boys		Girls	
	Model 1	Model 2	Model 1	Model 2
Prolonged sedentary bouts	0.001 (−0.129, 0.129)	0.001 (−0.128, 0.130)	0.066 (−0.035, 0.167)	0.056 (−0.046, 0.158)
Non-prolonged sedentary bouts	0.025 (−0.012, 0.061)	0.025 (−0.014, 0.064)	0.020 (−0.011, 0.051)	0.030 (−0.002, 0.063)
Breaks in ST	0.027 (−0.011, 0.064)	0.028 (−0.013, 0.068)	0.031 (−0.003, 0.064)	0.041 (0.007, 0.076) *
Duration of breaks in ST	0.010 (−0.004, 0.025)	0.011 (−0.004, 0.027)	0.015 (0.001, 0.028) *	0.021 (0.007, 0.035) **

Note: The values in the table are *B* (95% *CI*). Model 1 adjusted for age, urban and rural areas, SES, sleep time, and BMI; Model 2 adjusted for Model 1 + total ST. CRF, cardiorespiratory fitness; BMI, body mass index; SES, socioeconomic status; ST, sedentary time. * *p* < 0.05. ** *p* < 0.01.

**Table 6:** Exploration of the intensity attribute of the duration of breaks in ST in girls.

Independent Variables	Model 1	Model 2
Breaks in ST	0.075 (0.027, 0.123) **	0.003 (−0.042, 0.048)
Duration of breaks in ST	0.021 (0.012, 0.046) **	0.001 (−0.024, 0.025)

Note: The values in the table are *B* (95% *CI*). Model 1 adjusted for age, urban and rural areas, SES, sleep time, BMI, total sedentary time, and LPA; Model 2 adjusted for age, urban and rural areas, SES, sleep time, BMI, total sedentary time, and MVPA. CRF, cardiorespiratory fitness; BMI, body mass index; SES, socioeconomic status; ST, sedentary time. ** *p* < 0.01.

## Data Availability

The data that support the findings of this study are available from Xiaojian Yin but restrictions apply to the availability of these data, which were used under license for the current study and so are not publicly available.

## Supplementary Materials

The following supporting information can be downloaded at: https://www.mdpi.com/article/10.3390/children9081140/s1, Table S1: Association between screen time and CRF in girls. Table S2: Association between passive traffic ST and CRF in girls. Table S3: Association between breaks in ST and CRF in girls. Table S4: Association between duration of breaks in ST and CRF in girls.
